# Long-term evolution of Fe oxyhydroxide mineralogy and potentially toxic element partitioning in mining-impacted sediments of the Lower Doce River fluvial–estuarine system

**DOI:** 10.1007/s10653-026-03495-z

**Published:** 2026-09-21

**Authors:** Danilo de Lima Camêlo, Gabriel da Silva Abreu Souza, David Lukas de Arruda, Sara Ramos dos Santos, Poliana Borges de Oliveira, Maria Tereza Weitzel Dias Carneiro, Tiago Guimarães

**Affiliations:** 1 Graduate Program in Agrochemistry, Federal University of Espírito Santo, Alegre, Espírito Santo 29500-000 Brazil; 2 Graduate Program in Agronomy, Federal University of Espírito Santo, Alegre, Espírito Santo 29500-000 Brazil; 3 Graduate Program in Chemistry, Federal University of Espírito Santo, Vitória, Espírito Santo 29500-000 Brazil

**Keywords:** Multi-stressor environment, Isomorphic substitution, Preferential dissolution, Magnetic susceptibility, Trace-element partitioning

## Abstract

The collapse of the Fundão dam in southeastern Brazil released large quantities of Fe-rich tailings into the Doce River basin, generating a long-term legacy of mining-derived sediments enriched in potentially toxic elements (PTEs). Although Fe oxyhydroxides are recognized as major geochemical hosts of contaminants, their long-term mineralogical evolution and relationships with PTE partitioning in tropical fluvial–estuarine systems remain poorly understood. This study investigated the distribution, crystallographic properties, and geochemical behavior of Fe oxyhydroxides in sediments collected from reservoir and estuarine environments of the lower Doce River between 2019 and 2024. Selective dissolution procedures, X-ray diffraction, diffuse reflectance spectroscopy, magnetic susceptibility measurements, and multivariate analyses were used to characterize Fe oxyhydroxide assemblages and their relationships with the investigated PTEs. Crystalline Fe oxyhydroxides predominated throughout the study area, although temporal variations in Fe partitioning were consistent with ongoing mineralogical reorganization across contrasting depositional environments. Higher Fe_o_/Fe_d_ ratios at specific estuarine stations indicated relative enrichment of poorly crystalline Fe phases. Goethite remained the dominant Fe oxyhydroxide, whereas hematite exhibited greater spatial and temporal variability. Variations in crystal size, specific surface area, and Al substitution further suggest that Fe oxyhydroxide properties continue to evolve under present-day environmental conditions. Principal component analysis revealed strong associations between Fe oxyhydroxides and several PTEs, including As, Cr, Ni, Co, Pb, Zn, and Mo. The results indicate that Fe oxyhydroxides in mining-impacted sediments remain major geochemical hosts of the investigated PTEs more than a decade after the disaster, with their temporal variability closely associated with trace-element partitioning and potential redistribution. These findings highlight the importance of Fe mineralogy for understanding trace-element partitioning and potential redistribution and reinforce the value of mineralogical indicators and selective extraction procedures within long-term environmental monitoring frameworks.

## Highlights

1. Fe oxyhydroxides remain major geochemical hosts of the investigated PTEs in lower Doce River sediments.
2. Fe partitioning and Fe_o_/Fe_d_ ratios indicate ongoing mineralogical reorganization under estuarine forcing.
3. Goethite predominates throughout the system, while hematite exhibits greater spatial and temporal variability.
4. Crystallographic properties of Fe oxyhydroxides are strongly associated with the partitioning of the investigated PTEs.
5. Fe oxyhydroxides in mining-impacted sediments remain geochemically active more than a decade after the Fundão dam collapse.

## Introduction

The failure of the Fundão dam in November 2015 in Mariana, southeastern Brazil, released approximately 43 million m^3^ of Fe-rich mining tailings into the Doce River basin, constituting one of the largest mining-related environmental disasters worldwide (Carmo et al., 2017; Coimbra et al., 2019; Marta-Almeida et al., 2016). Fine-grained tailings (< 63 µm), composed predominantly of Fe oxyhydroxides and associated mineral phases, were transported more than 660 km downstream and deposited across river channels, floodplains, reservoirs, estuarine environments, and adjacent coastal ecosystems (Fernandes et al., 2016; Queiroz et al., 2018, 2022). This extensive dispersal introduced a persistent mineralogical and geochemical signature into the lower Doce River system, with long-term implications for sediment quality, contaminant dynamics, and ecosystem functioning (Camêlo et al., 2024; Leal Pacheco et al., 2025; Richard et al., 2020; Soares et al., 2025).

Iron oxyhydroxides, including goethite, hematite, ferrihydrite, and lepidocrocite, are among the most important reactive mineral phases in soils and sediments due to their high affinity for metals, metalloids, nutrients, and organic compounds (Camargo et al., 2018; Cornell & Schwertmann, 2003; Shi et al., 2021; von der Heyden & Roychoudhury, 2015). Through adsorption, co-precipitation, and structural incorporation mechanisms, these minerals exert strong control over contaminant mobility, bioavailability, and long-term geochemical cycling, particularly through interactions between Fe oxyhydroxide surfaces and organic matter (Dultz et al., 2018; Yi et al., 2022). Their reactivity is governed by mineralogical and crystallographic attributes, including crystallinity, particle size, specific surface area, crystal morphology, and isomorphic substitution, which collectively influence surface charge distribution, sorption site density, and dissolution kinetics (Dultz et al., 2018; Sun et al., 2023). In tropical environments, goethite and hematite commonly dominate Fe mineral assemblages owing to advanced weathering conditions, whereas poorly crystalline phases such as ferrihydrite and lepidocrocite become increasingly important under fluctuating redox regimes due to their high surface reactivity and lower thermodynamic stability (Cornell & Schwertmann, 2003; Kämpf & Schwertmann, 1983; Queiroz et al., 2022; Silva et al., 2020).

In the Doce River basin, elevated concentrations of potentially toxic elements (PTEs), including As, Cr, Mn, Pb, Zn, Cu, and Cd, have been reported in sediments, soils, and estuarine environments following the Fundão dam collapse, frequently exceeding regional background levels and environmental quality guidelines (Bertoldo et al., 2023; Gabriel et al., 2020; Hatje et al., 2017; Queiroz et al., 2018). Previous mineralogical investigations demonstrated that As and Pb enrichment may be closely associated with Al-rich goethite occurring in mining-impacted sediments, highlighting the role of Fe oxyhydroxides as major geochemical hosts of contaminants within the lower Doce River system (Camêlo et al., 2024). Seasonal flooding, sediment resuspension, organic matter degradation, and saltwater intrusion may, however, alter Fe oxyhydroxide stability and promote contaminant redistribution through dissolution–reprecipitation processes and changes in surface reactivity (Queiroz et al., 2018, 2022; Shi et al., 2021). Consequently, understanding the mineralogical evolution of Fe oxyhydroxides has become essential for predicting the long-term environmental behavior of contaminant-bearing sediments in the basin. In this study, consistent with our previous investigations of Fundão mine tailings and mining-impacted sediments of the Doce River basin (Arruda et al., 2025; Camêlo et al., 2024), the term potentially toxic elements (PTEs) refers specifically to the suite of trace elements analyzed (As, Cd, Co, Cr, Mo, Ni, Pb, V, and Zn) and does not imply a comprehensive assessment of all potentially hazardous elements occurring in mining-impacted sediments.

The stability of Fe oxyhydroxides is strongly influenced by environmental conditions capable of modifying their crystallographic properties (Cornell & Schwertmann, 2003). Variations in redox potential, pH, salinity, and hydrodynamic forcing may alter crystal growth patterns, mean crystal dimension, specific surface area, and the degree of isomorphic substitution, thereby affecting mineral reactivity and contaminant retention capacity (Cambier, 1986; Neumann et al., 2014; Queiroz et al., 2022). Reductive dissolution may destabilize crystalline and poorly crystalline Fe phases, whereas subsequent oxidation can promote the formation of short-range-order Fe oxyhydroxides characterized by elevated sorptive capacity and rapid geochemical turnover (Burdige & Komada, 2020; Zhang et al., 2019). Although the occurrence of Fe oxyhydroxides in sediments affected by the Fundão disaster has been documented, the extent to which their mineralogical and crystallographic properties evolve through time across contrasting depositional environments remains poorly constrained. In particular, little is known about how temporal changes in Fe oxyhydroxide mineralogical and crystallographic properties are related to PTE partitioning between Fe-bearing phases along the reservoir–estuary continuum of the lower Doce River.

This study was based on the hypothesis that Fe oxyhydroxides preserved in mining-impacted sediments undergo progressive mineralogical and crystallographic modification under seasonal hydrological variability, redox oscillations, and estuarine forcing, resulting in changes in PTE partitioning among Fe-bearing phases. Therefore, the objective of this study was to investigate the long-term evolution of Fe oxyhydroxide mineralogy and crystallographic properties in sediments of the lower Doce River fluvial–estuarine system and to evaluate their relationships with the partitioning of the investigated PTEs under contrasting reservoir and estuarine conditions. To address this objective, we integrated selective dissolution procedures, X-ray diffraction, diffuse reflectance spectroscopy, magnetic susceptibility measurements, crystallographic characterization, and multivariate statistical analyses to identify temporal mineralogical and geochemical patterns and infer process-based relationships between Fe oxyhydroxide reorganization and PTE partitioning in mining-impacted sediments.

## Materials and methods

### Study area and sampling

**Fig. 1:**
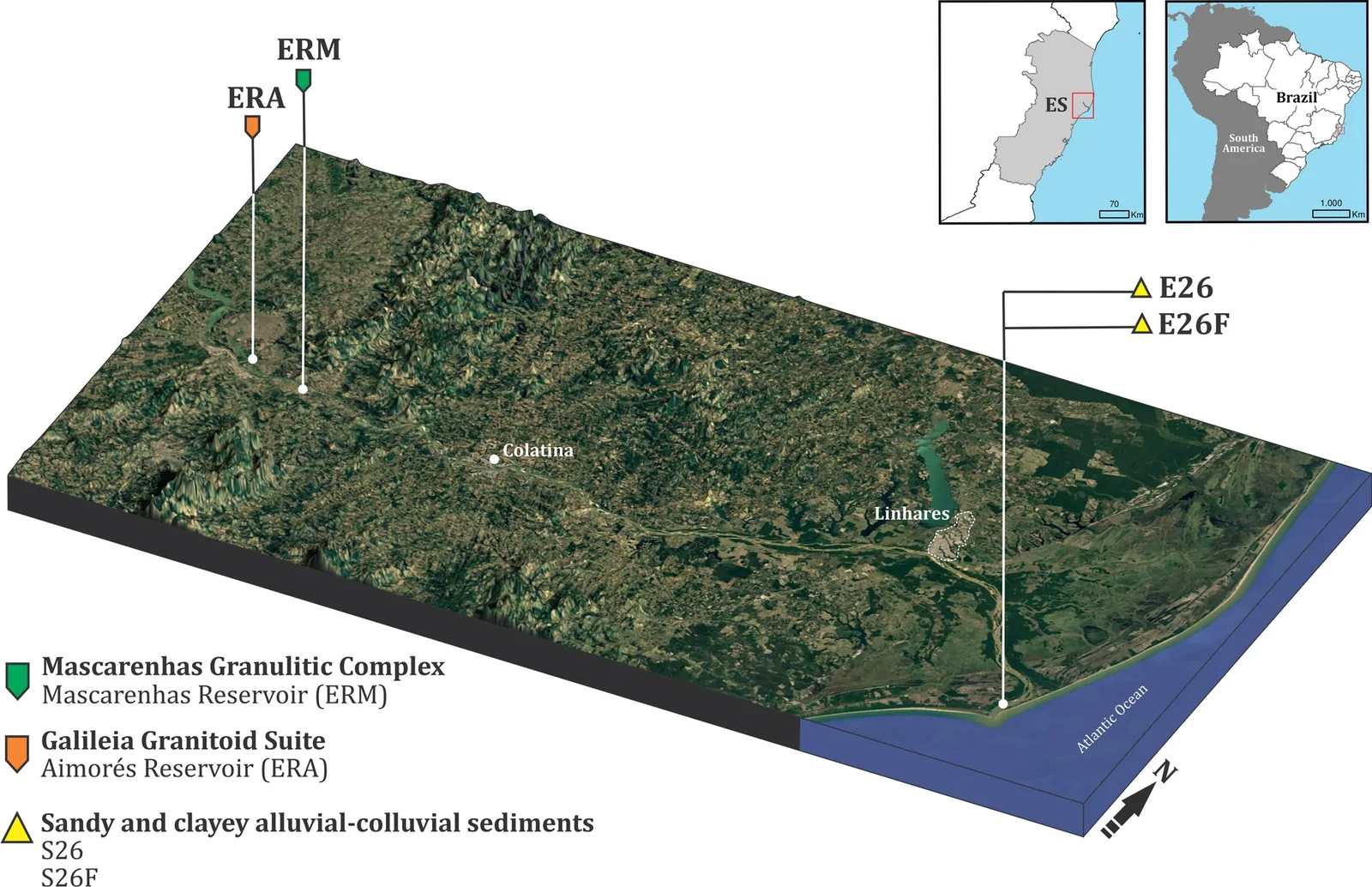
Study area and sampling locations along the lower Doce River fluvial–estuarine continuum, southeastern Brazil. Reservoir (ERA and ERM) and estuarine (E26 and E26F) environments represent contrasting geological, hydrodynamic, and sedimentary settings affected by the downstream transport and deposition of Fundão dam tailings. Monitoring was conducted between 2019 and 2024

The study area comprises the lower Doce River basin (Espírito Santo, Brazil), which was severely impacted by the failure of the Fundão tailings dam (Carmo et al., 2017; Coimbra et al., 2019). Four strategic sites were selected for bottom sediment sampling: The Aimorés Reservoir (ERA), located upstream (19°44'S, 41°18'W), lies within the geological context of the Galileia Granitoid Suite, a Precambrian batholith composed of tonalites, granodiorites, and granites. This site is characterized as a lentic environment with low hydrodynamic energy. Further downstream, the Mascarenhas Reservoir (ERM) (19°31'S, 40°54'W) is situated within the Mascarenhas Granulitic Complex, which is dominated by charnockitic and enderbithic rocks containing hypersthene. It represents a stable depositional environment with limited influence from hydrological pulses. At the Doce River mouth, the sampling sites E26 and E26F (19°38'S, 39°48'W) are in estuarine settings over sandy and clayey alluvial–colluvial sediments. Site E26F is subject to saltwater wedge intrusion, which promotes enhanced redox dynamics. The geographic location of all sites within the Doce River basin is shown in Fig. 1.

Sampling followed a spatiotemporal design to capture seasonal variations and post-disaster processes. Quarterly campaigns were conducted between March 2019 and July 2024, covering both dry (April-September) and wet (October–March) seasonal cycles. A total of 24 bottom-sediment samples were included in the analytical dataset of this study. At each sampling event, sediment was collected using a sterile Van Veen grab sampler, which retrieved a sediment sample about 30 cm deep. A 0.5-kg subsample was taken from the uppermost central portion of the grab (0–5 cm), placed in inert plastic bags, and stored at 4 °C until laboratory analysis. Sites E26 and E26F were monitored throughout the entire study period, whereas the reservoir sites ERA and ERM were incorporated into the monitoring program from August 2022 onward. Sites ERA and ERM represent low-energy sediment retention zones, suitable for assessing the accumulation of Fe oxyhydroxides and associated metals, whereas sites E26 and E26F monitor estuarine processes, including contaminant mobilization driven by saltwater intrusion and redox fluctuations.

### Laboratory analyses

#### Separation of the mud fraction

The bottom sediment samples were collected, freeze-dried, and homogenized. Subsequently, the mud fraction (< 63 µm) was separated from the sandy fraction by mechanical shaking at 50 rpm for 16 h, followed by particle-size separation through sieving. The resulting material was washed with deionized water and dried at 45 °C to preserve the reactive mineral phases (Camêlo et al., 2024).

#### Selective extractions and alkali fusion

The PTE suite investigated in this study comprised As, Cd, Co, Cr, Mo, Ni, Pb, V, and Zn. To operationally distinguish Fe fractions associated with different degrees of crystallinity, the following procedures were performed: (i) acid ammonium oxalate (AAO) extraction (0.2 mol L^−1^, pH 3.0, in the dark) to determine Fe_o_, Mn_o_, and associated PTEs, operationally representing fractions preferentially associated with lower-crystallinity Fe forms (e.g., ferrihydrite, lepidocrocite) (Mckeague & Day, 1966); (ii) Na–citrate–bicarbonate–dithionite (CBD) extraction at 60 °C to quantify Fe_d_, Mn_d_, and associated PTEs, operationally representing Fe-bearing fractions with a greater contribution from crystalline Fe oxyhydroxides (e.g., hematite, goethite, maghemite), following Mehra and Jackson (1958); and (iii) alkali fusion (AF) to determine total concentrations of Fe_t_, Mn_t_, and PTEs (Sulcek & Povondra, 1989). For alkali fusion, samples were thoroughly homogenized with a lithium borate flux (Maxxi Flux) containing 65 wt% lithium metaborate (LiBO_2_) and 35 wt% lithium tetraborate (Li_2_B_4_O_7_), and subsequently fused at 1050 °C for 30 min. The resulting glass beads were dissolved in 100 mL of 10% HCl prior to analysis. Iron and Mn concentrations in the CBD, AAO, and alkali fusion extracts were quantified by atomic absorption spectrometry (AAS), whereas PTE concentrations in these extracts were determined by inductively coupled plasma mass spectrometry (ICP-MS). These selective dissolution procedures provide operationally defined Fe and PTE fractions and were therefore interpreted as indicators of relative differences in Fe-phase crystallinity and associated trace-element partitioning, rather than as direct identification of specific mineral phases or element-binding mechanisms.

All analytical procedures followed rigorous quality control protocols to ensure analytical accuracy and reproducibility. Certified reference materials (SQC001—Metals in Soil) were used to validate both selective extraction and alkali fusion procedures. Recoveries for major and trace elements ranged from 93 to 109%. Procedural blanks were included in each analytical batch to monitor potential contamination. Limits of detection, calculated as three times the standard deviation of blank replicates, ranged from 0.01 to 2 mg kg^−1^ for trace elements. Duplicate samples (n = 20% of total samples) exhibited relative standard deviations below 10% for Fe, Mn, and PTE determinations, indicating satisfactory analytical precision and reproducibility.

#### X-ray diffraction (XRD) and crystallographic parameters

To investigate the crystallographic parameters of Fe oxyhydroxides, the samples were subjected to alkaline digestion using a 5 mol L^−1^ NaOH solution to remove silicates and concentrate the crystalline Fe oxyhydroxides (Norrish & Taylor, 1961). Mineralogical characterization was performed by X-ray diffraction (XRD; Rigaku Miniflex 600C) using CuKα radiation operated at 40 kV and 15 mA, with data collected in continuous scan mode. For bulk sediment samples (< 63 µm; mud fraction), XRD analyses were performed over an angular range of 4–70°2θ, using a scan speed of 1.2°2θ min^−1^ and a step size of 0.02°2θ. For Fe oxyhydroxide concentrates, the angular range was restricted to 4–55°2θ, with a slower scan speed of 0.4°2θ min^−1^, while maintaining the same step size. Mineral identification was based on the position and relative intensity of the characteristic diffraction peaks.

Crystallographic parameters were calculated as follows. The mean crystal dimension (MCD) was obtained using the Scherrer equation corrected for Bragg angles (Melo et al., 2001). The specific surface area (SSA) and aluminum isomorphic substitution (IS) were then estimated according to Kämpf and Schwertmann (1983) and Schulze (1984), respectively. The specific surface area (SSA) of goethite and hematite was estimated following Kämpf and Schwertmann (1983). For goethite, SSA was calculated as SSA_Gt_ = (1049 / MCD_100_) − 5 (m^2^ g^−1^), where MCD_100_ corresponds to the MCD of the 100 plane, given by MCD_100_ = d_110_ × 0.42 nm. For hematite, SSA was determined according to SSA_Hm_ = 2 × (r + h) × 10^3^ / (r × h × d) (m^2^ g^−1^), where r = MCD_110_ × 0.71 / 2, h = MCD_012_ × 0.59, and d = 5.26 g cm^−3^.

The Al substitution in the hematite (IS_Hm_) and goethite (IS_Gt_) structures was estimated from XRD data according to Schulze (1984). The IS_Hm_ was calculated as Al (mol mol^−1^) = 31.09 − 6.17 × a_0_, where a_0_ represents the unit-cell parameter derived from the d_110_ diffraction peak, according to a_0_ = 2 × d_110_. The IS_Gt_ was estimated as Al (mol mol^−1^) = 17.30 − 5.72 × c_0_, where c_0_ is the unit-cell parameter calculated from the d_110_ and d_111_ diffraction peaks using c_0_ = [(1/d_111_^2^) − (1/d_110_^2^)]^−0.5^.

Although alkaline digestion (pretreatment with 5 mol L^−1^ NaOH) substantially reduces interference from silicate phases and associated peak overlap, uncertainties related to microstrain, structural disorder, and peak broadening cannot be completely excluded. Therefore, MCD, SSA, and IS estimates were interpreted primarily as comparative crystallographic descriptors among sampling sites and monitoring campaigns rather than as absolute quantitative measurements.

#### Magnetic susceptibility (χ)

Mass-specific magnetic susceptibility (χ) was measured in 10 cm^3^ aliquots of mud fraction (< 63 µm) using a Bartington MS3 system coupled to the MS2B sensor at low (χ_lf_; 0.47 kHz) and high frequencies (χ_hf_; 4.70 kHz). Frequency-dependent susceptibility (χ_fd_) was calculated as: χ_fd_ = 100 × [(χ_lf_ – χ_hf_) / χ_lf_] (Dearing, 1999).

#### Diffuse reflectance spectroscopy (DRS)

Diffuse reflectance spectroscopy was carried out using a PerkinElmer Lambda 365+ UV/Vis spectrophotometer equipped with a 50 mm integrating sphere with a Spectralon® (PTFE-based) diffuse reflectance reference standard. Reflectance data acquired over the spectral range of 380–710 nm, with a slit width of 1 nm, data interval of 0.5 nm, and scan speed of 2 nm s^−1^, were transformed according to the Kubelka–Munk function (Kubelka & Munk, 1931), and its second derivative was used to enhance the diagnostic absorption features of goethite (Gt) and hematite (Hm). Band positions, integrated peak areas, and the ratios Gt/(Gt + Hm) and Hm/(Gt + Hm) were used to estimate the relative abundance of goethite and hematite and, in combination with selectively extracted Fe concentrations, to estimate the contribution of these dominant crystalline Fe oxyhydroxides (Cao et al., 2022). DRS was used as a complementary approach to assess the relative contribution of goethite and hematite, providing sensitive diagnostic spectral features when XRD reflections are weak or affected by mineralogical complexity. DRS data were interpreted jointly with XRD and selective dissolution results and were not used to resolve poorly crystalline phases or short-range structural ordering.

#### Statistical analyses

Principal component analysis (PCA) and hierarchical cluster analysis (HCA) were performed to evaluate relationships among total and selectively extracted Fe fractions, Fe oxyhydroxide crystallographic properties, magnetic susceptibility, and PTEs. Prior to analysis, all variables were mean-centered and scaled to unit variance. Statistical analyses were conducted using R (v4.6.0) in the RStudio environment (R Core Team, 2026).

## Results

### Total concentrations of the investigated potentially toxic elements (PTEs)

Total concentrations of the investigated PTEs in the sediment mud fraction (< 63 µm) showed marked differences among sampling environments and monitoring periods along the lower Doce River system (Table 1). In general, estuarine stations (E26 and E26F) consistently exhibited the highest concentrations of most analyzed elements, particularly As, Cr, Ni, and Zn, whereas the reservoir environments (ERA and ERM) presented comparatively lower concentrations.

**Table 1:** Total concentrations of the investigated potentially toxic elements (PTEs) in the sediment mud fraction (< 63 µm) determined by alkaline fusion digestion followed by ICP-MS analysis

Station	Campaign	Period	Date	As	Cd	Co	Cr	Mo	Ni	Pb	V	Zn
				g kg^−1^								
E26	C7	Dry	Apr/19	21.90	1.65	16.09	138.44	5.17	266.06	13.44	104.98	73.58
E26	C8	Dry	May/19	21.97	1.43	14.21	122.29	5.03	240.86	11.72	104.03	81.69
E26	C10	Dry	Jul/19	22.74	1.70	17.33	125.79	5.09	251.23	13.15	101.17	74.57
E26	C53	Wet	Oct/23	37.86	1.58	27.60	128.01	3.73	282.60	14.64	88.14	105.34
E26	C54	Wet	Nov/23	47.20	1.47	33.27	154.46	3.64	300.30	15.53	88.66	90.59
E26	C57	We	Dec/23	35.74	1.24	27.11	134.08	4.23	294.75	15.75	94.53	81.12
E26	C60	Wet	Mar/24	45.20	1.48	45.02	148.60	4.24	331.67	16.87	106.39	136.56
E26F	C45	Dry	Ago/22	42.63	1.83	17.57	129.94	4.99	310.51	47.75	115.97	62.13
E26F	C46	Wet	Oct/22	36.13	1.67	17.90	139.87	8.62	264.28	17.31	91.59	50.58
E26F	C48	Wet	Dec/22	50.35	1.61	27.79	170.91	6.69	364.02	102.15	120.54	90.59
E26F	C51	Wet	Mar/23	50.03	1.76	25.88	321.04	23.37	416.15	36.90	100.61	241.75
E26F	C57	Wet	Dec/23	23.83	1.20	19.40	107.92	3.86	230.13	10.80	96.39	74.00
E26F	C60	Wet	Mar/24	64.26	1.62	39.62	275.05	20.90	448.33	33.50	120.97	124.93
ERA	C45	Dry	Ago/22	18.10	1.07	16.71	119.28	3.36	276.50	10.66	94.49	49.08
ERA	C46	Wet	Oct/22	7.36	1.19	18.61	48.51	3.93	197.86	7.15	114.50	133.44
ERA	C47	Wet	Nov/22	20.81	1.17	13.40	102.05	3.46	244.80	15.08	91.09	396.46
ERA	C51	Wet	Mar/23	9.22	1.22	18.82	45.58	3.63	176.64	7.05	109.62	117.64
ERA	C53	Dry	Jul/23	9.29	1.17	17.63	51.01	3.62	193.93	6.47	104.09	111.99
ERA	C55	Wet	Oct/23	8.19	1.12	19.19	40.57	3.70	185.40	5.75	111.85	70.72
ERA	C57	Wet	Dec/23	8.19	1.15	18.20	43.43	3.83	199.84	6.23	122.12	94.63
ERM	C45	Dry	Ago/22	24.35	1.64	19.31	154.39	9.52	262.34	18.52	97.69	100.77
ERM	C55	Wet	Oct/23	22.29	1.53	17.33	106.70	3.96	234.24	12.76	101.37	60.24
ERM	C57	Wet	Dec/23	16.53	1.55	15.96	107.06	4.00	215.21	11.58	95.18	56.85
ERM	C60	Wet	Mar/24	28.79	1.62	22.74	110.35	3.86	228.51	11.35	97.43	61.34

Among the analyzed elements, Ni showed the highest concentrations throughout the study area, reaching 448.33 mg kg^−1^ at E26F during Mar/24, followed by Cr, which reached 321.04 mg kg^−1^ at the same station during Mar/23. Arsenic concentrations also exhibited marked enrichment at E26F, varying from 23.83 to 64.26 mg kg^−1^, while E26 showed progressively higher As concentrations between 2019 and 2024. In contrast, ERA generally exhibited the lowest concentrations of As and Cr, although localized enrichment of Zn was observed during some campaigns, particularly in Nov/22, when concentrations reached 396.46 mg kg^−1^.

Temporal variations were more pronounced at the estuarine stations. At E26F, increases in Co, Cr, Mo, Ni, and Zn were particularly evident during wet-period campaigns, whereas E26 exhibited comparatively moderate but persistent enrichment trends over time. Reservoir stations displayed lower temporal variability overall, although fluctuations in Zn and Cr concentrations were observed at ERA between wet and dry periods. Collectively, the results indicate persistent enrichment of Fe-associated PTEs within the estuarine sector relative to the upstream reservoir environments.

### Distribution of PTEs in selective dissolution fractions and AAO/CBD ratios

Selective dissolution analyses using CBD and AAO revealed distinct distribution patterns of PTEs among operationally defined Fe-bearing fractions across the monitored environments and sampling campaigns (Tables 2 and 3). In general, CBD-extractable concentrations were consistently higher than AAO-extractable concentrations for most analyzed elements, indicating that a substantial proportion of the PTEs was associated with the CBD-extractable Fe fraction, operationally related to more crystalline Fe oxyhydroxide forms.

**Table 2:** Concentrations of the investigated potentially toxic elements (PTEs) in the sediment mud fraction (< 63 µm) obtained by selective dissolution using Na–citrate–bicarbonate–dithionite (CBD) and acid ammonium oxalate (AAO) extractants

Station	Campaign	Period	Date	As	Cd	Co	Cr	Mo	Ni	Pb	V	Zn
				g kg^−1^								
				AAO extractant								
E26	C6	Wet	Mar/19	1.85	0.05	2.06	6.40	0.35	15.19	0.54	8.30	23.52
E26	C9	Dry	Jun/19	1.95	0.04	1.91	5.99	0.25	13.19	0.45	6.88	4.10
E26	C54	Dry	Sep/23	6.01	0.06	9.19	12.25	0.32	34.62	1.46	14.61	14.00
E26	C57	Wet	Dec/23	4.82	0.05	6.02	8.98	0.47	30.45	1.13	15.08	7.40
E26F	C47	Wet	Nov/22	6.42	0.06	5.92	14.28	0.51	59.07	0.73	29.13	13.56
E26F	C52	Dry	May/23	6.76	0.06	9.31	12.80	0.61	61.39	1.01	30.45	17.49
E26F	C57	Wet	Dec/23	1.01	0.05	0.89	2.67	0.20	7.10	0.36	2.87	4.48
ERA	C46	Wet	Nov/22	1.30	0.05	8.44	3.78	0.38	35.69	0.60	28.01	12.74
ERA	C51	Wet	Mar/23	1.22	0.05	8.61	4.57	0.39	37.39	0.74	33.13	11.09
ERA	C53	Dry	Jul/23	1.40	0.06	7.46	4.13	0.31	41.38	0.56	25.80	19.10
ERA	C57	Wet	Dec/23	1.19	0.05	7.38	3.46	0.29	34.79	0.54	23.50	12.67
ERM	C46	Wet	Agu/22	2.32	0.06	4.24	6.58	0.49	27.58	0.63	13.10	6.39
ERM	C54	Dry	Sep/23	1.99	0.04	5.84	7.62	0.29	34.61	0.64	18.88	11.03
ERM	C57	Wet	Dec/23	2.60	0.05	2.76	8.38	0.41	33.68	0.57	19.57	0.66
				CBD extractant								
E26	C6	Wet	Mar/19	5.72	0.17	3.30	18.31	1.11	55.69	0.93	26.25	0.70
E26	C9	Dry	Jun/19	5.99	0.18	3.38	18.57	1.04	57.72	0.98	27.43	0.14
E26	C54	Dry	Sep/23	34.38	0.26	18.59	53.82	1.54	162.72	3.82	51.28	45.33
E26	C57	Wet	Dec/23	23.47	0.19	13.11	43.01	1.57	133.03	2.59	48.08	23.10
E26F	C47	Wet	Nov/22	12.22	0.18	6.20	35.98	1.45	120.77	1.43	61.19	10.31
E26F	C52	Dry	May/23	7.47	0.19	3.56	17.11	1.34	58.20	1.13	25.93	15.07
E26F	C57	Wet	Dec/23	14.81	0.18	10.38	36.23	1.71	130.46	1.73	62.44	18.25
ERA	C46	Wet	Nov/22	2.91	0.19	7.85	5.94	0.98	52.16	0.83	35.08	5.35
ERA	C51	Wet	Mar/23	3.26	0.18	7.67	5.74	1.02	45.58	0.85	37.69	0.53
ERA	C53	Dry	Jul/23	3.71	0.18	8.48	11.00	1.14	72.35	0.91	42.33	13.72
ERA	C57	Wet	Dec/23	3.21	0.21	7.51	7.29	1.03	58.25	0.85	36.38	4.86
ERM	C46	Wet	Agu/22	7.47	0.19	8.09	31.11	1.56	102.98	1.45	47.90	11.39
ERM	C54	Dry	Sep/23	6.54	0.20	6.72	35.25	1.31	114.52	1.27	57.67	13.70
ERM	C57	Wet	Dec/23	6.16	0.22	6.47	31.09	1.29	102.83	1.24	53.35	11.71

**Table 3:** Ratios between AAO- and CBD-extractable investigated potentially toxic elements (PTEs) in the sediment mud fraction (< 63 µm)

Station	Campaign	Period	Date	As	Cd	Co	Cr	Mo	Ni	Pb	V	Zn
				Non-dimensional								
E26	C6	Wet	Mar/19	0.32	0.31	0.63	0.35	0.32	0.27	0.58	0.32	33.70
E26	C9	Dry	Jun/19	0.33	0.23	0.57	0.32	0.24	0.23	0.46	0.25	29.73
E26	C54	Dry	Sep/23	0.17	0.22	0.49	0.23	0.21	0.21	0.38	0.28	0.31
E26	C57	Wet	Dec/23	0.21	0.28	0.46	0.21	0.30	0.23	0.44	0.31	0.32
E26F	C47	Wet	Nov/22	0.53	0.33	0.96	0.40	0.35	0.49	0.51	0.48	1.32
E26F	C52	Dry	May/23	0.90	0.31	2.61	0.75	0.46	1.05	0.89	1.17	1.16
E26F	C57	Wet	Dec/23	0.07	0.30	0.09	0.07	0.12	0.05	0.21	0.05	0.25
ERA	C46	Wet	Nov/22	0.45	0.27	1.08	0.64	0.39	0.68	0.72	0.80	2.38
ERA	C51	Wet	Mar/23	0.38	0.27	1.12	0.80	0.38	0.82	0.87	0.88	21.00
ERA	C53	Dry	Jul/23	0.38	0.31	0.88	0.38	0.27	0.57	0.61	0.61	1.39
ERA	C57	Wet	Dec/23	0.37	0.23	0.98	0.48	0.28	0.60	0.63	0.65	2.61
ERM	C46	Wet	Agu/22	0.31	0.30	0.52	0.21	0.32	0.27	0.43	0.27	0.56
ERM	C54	Dry	Sep/23	0.30	0.22	0.87	0.22	0.22	0.30	0.50	0.33	0.81
ERM	C57	Wet	Dec/23	0.42	0.21	0.43	0.27	0.31	0.33	0.46	0.37	0.06

Estuarine stations exhibited the highest concentrations in both extractable fractions. At E26, CBD-extractable As increased from 5.72 mg kg^−1^ in Mar/19 to 34.38 mg kg^−1^ in Sep/23, accompanied by marked increases in Cr, Ni, and V. Similarly, E26F exhibited elevated CBD-extractable concentrations of Ni, Cr, and V, particularly during wet-period campaigns, with Ni reaching 130.46 mg kg^−1^ in Dec/23. In contrast, reservoir stations generally presented lower As concentrations but relatively elevated concentrations of Co, Ni, and V in both extractable fractions.

The AAO-extractable fractions showed comparatively lower concentrations and greater temporal variability than the CBD fractions. At E26F, AAO-extractable Ni exceeded 60 mg kg^−1^ during Nov/22 and May/23 but decreased markedly during Dec/23. Similar oscillations were observed for As, Co, Cr, and V. Reservoir stations, particularly ERA, exhibited comparatively stable AAO-extractable concentrations throughout the monitoring period.

The AAO/CBD ratios further revealed marked differences in the relative distribution of PTEs between operationally defined Fe fractions associated with lower and higher degrees of crystallinity (Table 3). Lower ratios were generally observed for As, Cr, Mo, and Ni, particularly at E26 and ERM, indicating greater relative association with the CBD-extractable fraction. In contrast, comparatively higher ratios were observed for Co, V, and Zn at specific stations and sampling periods, with particularly high Zn ratios at E26 during 2019 and elevated ratios for Co, Ni, V, and Zn at E26F and ERA.

At E26F during May/23, Co, Ni, and V exhibited AAO/CBD ratios ≥ 1.0, indicating relative enrichment within the AAO-extractable fraction during this campaign. Conversely, the lowest ratios for most elements were recorded at E26F during Dec/23, reflecting a marked reduction in the relative contribution of the AAO-extractable fraction during this period. Overall, the AAO/CBD ratios indicate distinct partitioning patterns of PTEs between operationally defined Fe-bearing fractions with contrasting degrees of crystallinity along the fluvial–estuarine continuum, with estuarine stations exhibiting the greatest temporal variability.

### Temporal variability of Fe oxyhydroxide phases and crystallographic properties

**Fig. 2:**
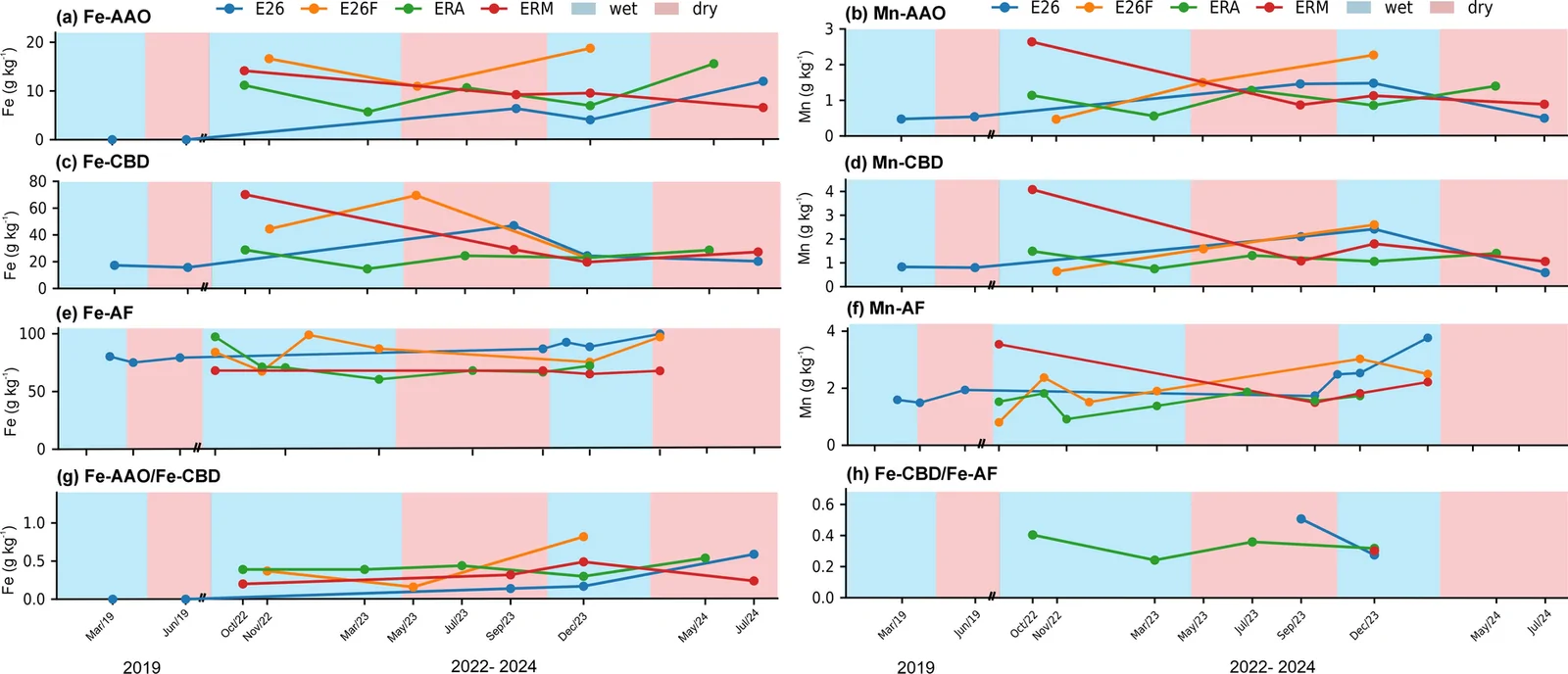
Seasonal and spatial dynamics of Fe and Mn associated with Fe oxyhydroxide fractions in sediments of the lower Doce River basin. Variations in AAO-extractable Fe (**a**) and Mn (**b**), CBD-extractable Fe (**c**) and Mn (**d**), total (alkali fusion – AF) Fe (**e**) and Mn (**f**), and the Fe-AAO/Fe-CBD (Fe_o_/Fe_d_) (**g**) and Fe-CBD/Fe-AF (Fe_d_/Fe_t_) (**h**) ratios illustrate changes in the relative contribution of operationally defined Fe fractions with contrasting degrees of crystallinity under different hydrological conditions. Blue and red shaded areas indicate wet and dry periods, respectively

Crystalline Fe oxyhydroxides predominated throughout the monitoring period, as indicated by consistently higher CBD-extractable Fe concentrations (Fe_d_) relative to AAO-extractable Fe concentrations (Fe_o_) across all sampling environments (Fig. 2). Fe_d_ concentrations ranged from approximately 15 to 70 g kg^−1^, with the highest values recorded at ERM and E26F, whereas Fe_o_ concentrations remained below 20 g kg^−1^. Total Fe concentrations (< 95 g kg^–1^) determined by alkali fusion (Fe_t_) exhibited low temporal variability and consistently higher values at E26 and E26F.

The Fe_o_/Fe_d_ ratio ranged from 0.14 to 0.82 and varied markedly among environments and sampling campaigns. Higher Fe_o_/Fe_d_ values occurred during specific monitoring campaigns, particularly at E26 and E26F. These stations also exhibited markedly lower ratios from late 2023 to early 2024, highlighting their pronounced temporal variability. Estuarine stations generally exhibited greater temporal fluctuations in Fe_o_/Fe_d_ than the reservoir environments. The Fe_d_/Fe_t_ ratio varied from approximately 0.25 to 0.50, with elevated values observed at the estuarine stations, particularly during late 2023.

Manganese concentrations displayed greater temporal variability than Fe in both AAO- and CBD-extractable fractions. AAO-extractable Mn (Mn_o_) concentrations ranged from 0.47 to 2.64 g kg^−1^, whereas CBD-extractable Mn (Mn_d_) varied from 0.64 to 4.08 g kg^−1^. The highest Mn_d_ values occurred at ERM during Oct/22. Total Mn concentrations (< 3.05 g kg^–1^) obtained by alkali fusion (Mn_t_) also fluctuated substantially among sampling periods, particularly at the estuarine stations. Overall, Mn exhibited greater temporal variability than Fe, whereas Fe remained predominantly associated with the CBD-extractable fraction throughout the monitoring period.

**Fig. 3:**
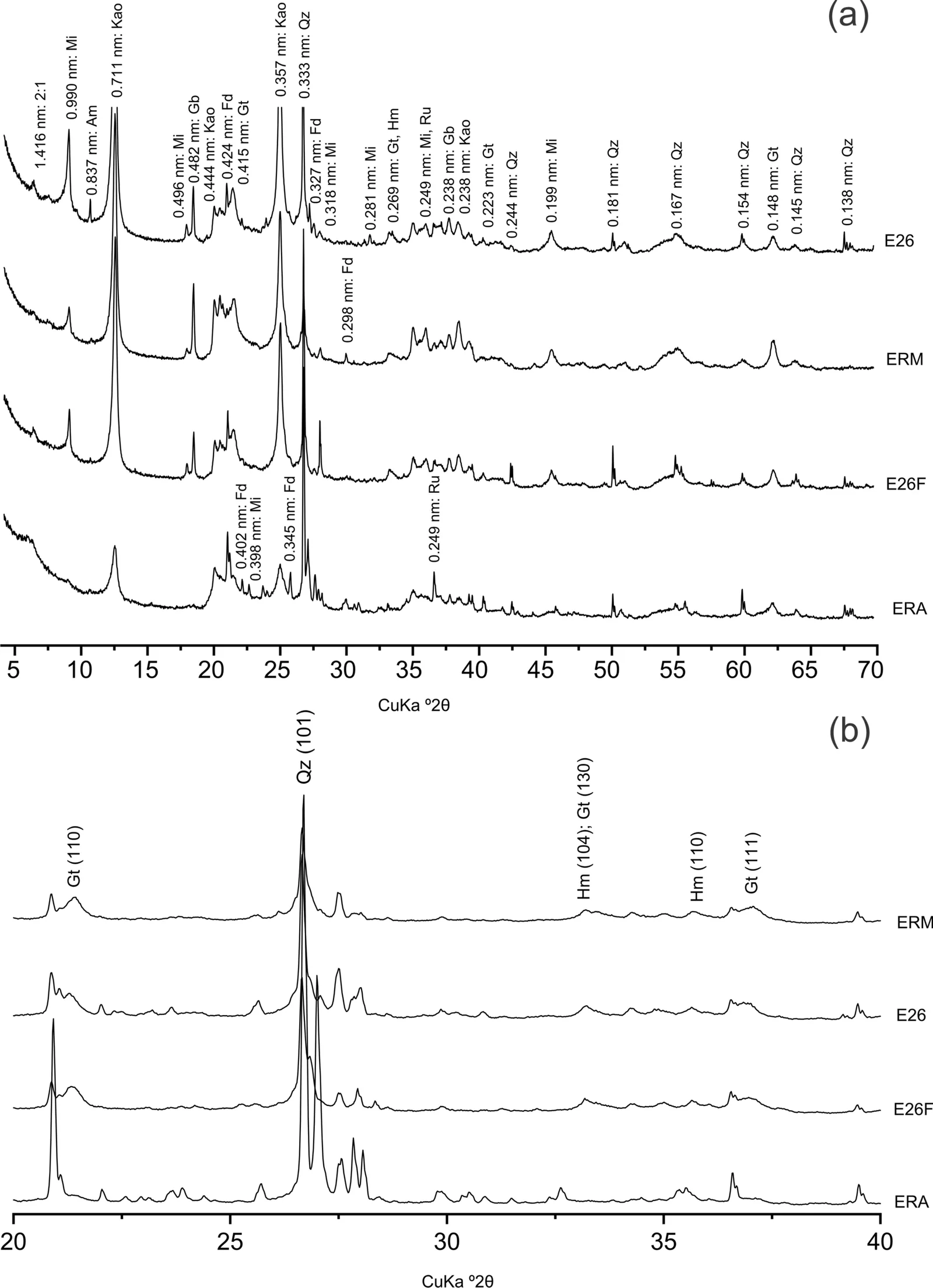
X-ray diffraction patterns of the natural mud fraction (< 63 µm) (**a**) and Fe oxyhydroxide concentrates obtained after treatment with 5 mol L^−1^ NaOH (**b**) from fluvial–estuarine sediments collected at different monitoring stations in the lower Doce River basin. 2:1 Clay = 2:1 clay mineral; Mi = mica (muscovite); Fd = K-feldspar, kao = kaolinite; Qz = quartz; Gb = gibbsite; Ru = rutile; Gt = goethite; Hm = hematite. Values in nanometers (nm) correspond to d-spacing

The X-ray diffraction patterns revealed a relatively homogeneous mineralogical composition across the monitored environments and sampling periods (Fig. 3). Quartz was the dominant mineral phase in all samples, accompanied by K-feldspar, 2:1 clay minerals, kaolinite, gibbsite, muscovite, goethite, hematite, and rutile. Variations in peak intensities were observed among sites and campaigns, particularly for Fe oxyhydroxides, although no substantial differences in the overall mineral assemblage were detected throughout the monitoring period. The Fe oxyhydroxide concentrates confirmed the predominance of goethite throughout the monitored system, whereas hematite occurred as a subordinate phase and was more evident in sediments from E26, E26F, and ERM. In contrast, Fe oxyhydroxide reflections were comparatively less intense in ERA sediments.

The predominance of crystalline Fe forms indicated by the selective dissolution data was consistent with the mineralogical composition of the Fe oxyhydroxide concentrates (Table 4 and Fig. 3). Goethite was identified in all samples, with d-spacing values for the Gt_110_ reflection ranging from 0.415 to 0.417 nm. Hematite was detected in most samples from E26, E26F, and ERM, whereas it was not identified in the ERA samples, indicating differences in Fe oxyhydroxide composition between the estuarine and reservoir environments.

**Table 4:** Crystallographic parameters of Fe oxyhydroxides in mud sediment concentrates, including d-spacing (d), mean crystal dimension (MCD), specific surface area (SSA), and Al-for-Fe isomorphic substitution in goethite (IS_Gt_) and hematite (IS_Hm_), together with mass-specific low-frequency (χ_lf_) and frequency-dependent (χ_fd_) magnetic susceptibility values obtained from the mud fraction sediments

Station	Campaign	Period	Date	d-spacing (nm)				MCD (nm)			
				Gt_110_	Gt_111_	Hm_104_	Hm_110_	Gt_110_	Gt_111_	Hm_104_	Hm_110_
E26	C6	Wet	Mar/19	0.417	0.243	0.270	0.252	22.36	17.68	38.23	30.30
E26	C9	Dry	Jun/19	0.417	0.243	0.270	0.252	22.27	13.96	26.99	37.67
E26	C54	Dry	Sep/23	0.417	0.244	0.270	0.252	40.26	18.33	42.48	30.89
E26	C57	Wet	Dec/23	0.417	0.244	0.270	0.252	21.57	15.66	50.84	65.29
E26	C62	Dry	Jul/24	0.416	0.243	0.269	0.252	22.03	15.39	58.45	63.39
E26F	C47	Wet	Nov/22	0.416	0.243	0.270	0.252	23.73	13.69	35.49	75.98
E26F	C52	Dry	May/23	0.417	0.244	0.270	0.251	22.38	14.53	56.86	43.75
E26F	C57	Wet	Dec/23	0.416	0.244	0.270	0.251	20.06	11.91	32.86	28.10
ERA	C46	Wet	Oct/22	0.417	0.244	n.d	n.d	23.23	21.23	n.d	n.d
ERA	C51	Wet	Mar/23	0.416	0.243	n.d	n.d	31.02	15.85	n.d	n.d
ERA	C53	Dry	Jul/23	0.416	0.243	n.d	n.d	26.11	17.90	n.d	n.d
ERA	C57	Wet	Dec/23	0.416	0.243	n.d	n.d	26.32	18.05	n.d	n.d
ERA	C61	Dry	May/24	0.416	0.244	n.d	n.d	32.79	15.39	n.d	n.d
ERM	C46	Wet	Oct/22	0.416	0.243	0.270	0.252	21.48	14.06	29.36	30.58
ERM	C54	Dry	Sep/23	0.415	0.243	0.270	0.252	35.73	15.53	33.88	23.12
ERM	C57	Wet	Dec/23	0.416	0.243	0.270	0.252	25.47	12.83	31.98	23.82
ERM	C62	Dry	Jul/24	0.415	0.243	0.270	0.251	26.57	17.99	56.91	38.62

Station	Campaign	Period	Date	MCD_Gt110_/	MCD_Hm104_/	SSA_Gt_	IS_Gt_	IS_Hm_	χ_lf_	χ_fd_
				MCD_Gt111_	MCD_Hm110_	(m^2^ g^−1^)	mol mol^−1^		10^–6^ m^3^ kg^−1^	%
E26	C6	Wet	Mar/19	1.26	1.26	106.69	0.190	0.025	–	–
E26	C9	Dry	Jun/19	1.60	0.72	107.17	0.161	0.010	–	–
E26	C54	Dry	Sep/23	2.20	1.38	57.03	0.099	0.029	1.84	3.1
E26	C57	Wet	Dec/23	1.38	0.78	110.81	0.127	0.017	–	–
E26	C62	Dry	Jul/24	1.43	0.92	108.39	0.116	0.044	–	–
E26F	C47	Wet	Nov/22	1.73	0.47	100.26	0.196	0.004	0.80	5.1
E26F	C52	Dry	May/23	1.54	1.30	106.59	0.097	0.071	0.83	8.1
E26F	C57	Wet	Dec/23	1.68	1.17	119.51	0.110	0.067	0.81	9.4
ERA	C46	Wet	Oct/22	1.09	n.d	102.50	0.125	n.d	1.38	8.2
ERA	C51	Wet	Mar/23	1.96	n.d	75.51	0.144	n.d	0.51	5.7
ERA	C53	Dry	Jul/23	1.46	n.d	90.66	0.163	n.d	0.63	6.6
ERA	C57	Wet	Dec/23	1.46	n.d	89.88	0.141	n.d	0.87	7.2
ERA	C61	Dry	May/24	2.13	n.d	71.18	0.110	n.d	0.75	7.7
ERM	C46	Wet	Oct/22	1.53	0.96	111.28	0.150	0.004	–	–
ERM	C54	Dry	Sep/23	2.30	1.47	64.91	0.121	0.021	0.77	10.7
ERM	C57	Wet	Dec/23	1.98	1.34	93.06	0.167	0.042	0.72	5.0
ERM	C62	Dry	Jul/24	1.48	1.47	89.01	0.199	0.054	–	–

n.d. = not detected; – = not determined

The mean crystal dimension of goethite along the [110] direction (MCD_Gt110_) ranged from 20.06 to 40.26 nm, whereas MCD_Gt111_ varied from 11.91 to 21.23 nm. The MCD_Gt110_/MCD_Gt111_ ratio remained consistently higher than 1.0 in all samples, indicating preferential crystal growth along the [110] direction. Particularly high ratios (> 2.0) were observed at ERM (2.30; Sep/23), E26 (2.20; Sep/23), and ERA (2.13; May/24), predominantly during dry-period campaigns. These elevated ratios suggest the occurrence of elongated goethite crystals and indicate marked variability in crystal morphology among sampling environments and periods.

The specific surface area of goethite (SSA_Gt_) ranged from 57.03 to 119.51 m^2^ g^−1^, with the lowest value recorded at E26 during the dry period of 2023 and the highest at E26F during the wet period of 2023. The IS_Gt_ values ranged from 0.097 to 0.199 mol mol^−1^, indicating considerable variation in Al substitution among environments and monitoring periods. These variations suggest differences in goethite crystallinity, crystal growth conditions, and surface reactivity across the monitored system.

Hematite exhibited comparatively larger crystallite dimensions than goethite, with MCD_Hm104_ ranging from 26.99 to 58.45 nm and MCD_Hm110_ varying from 23.12 to 75.98 nm. Greater variability in hematite crystallinity was observed at the estuarine stations, particularly at E26 and E26F. In contrast, the isomorphic substitution of Fe by Al in hematite (IS_Hm_) remained comparatively low and less variable, ranging from 0.004 to 0.071 mol mol^−1^.

**Fig. 4:**
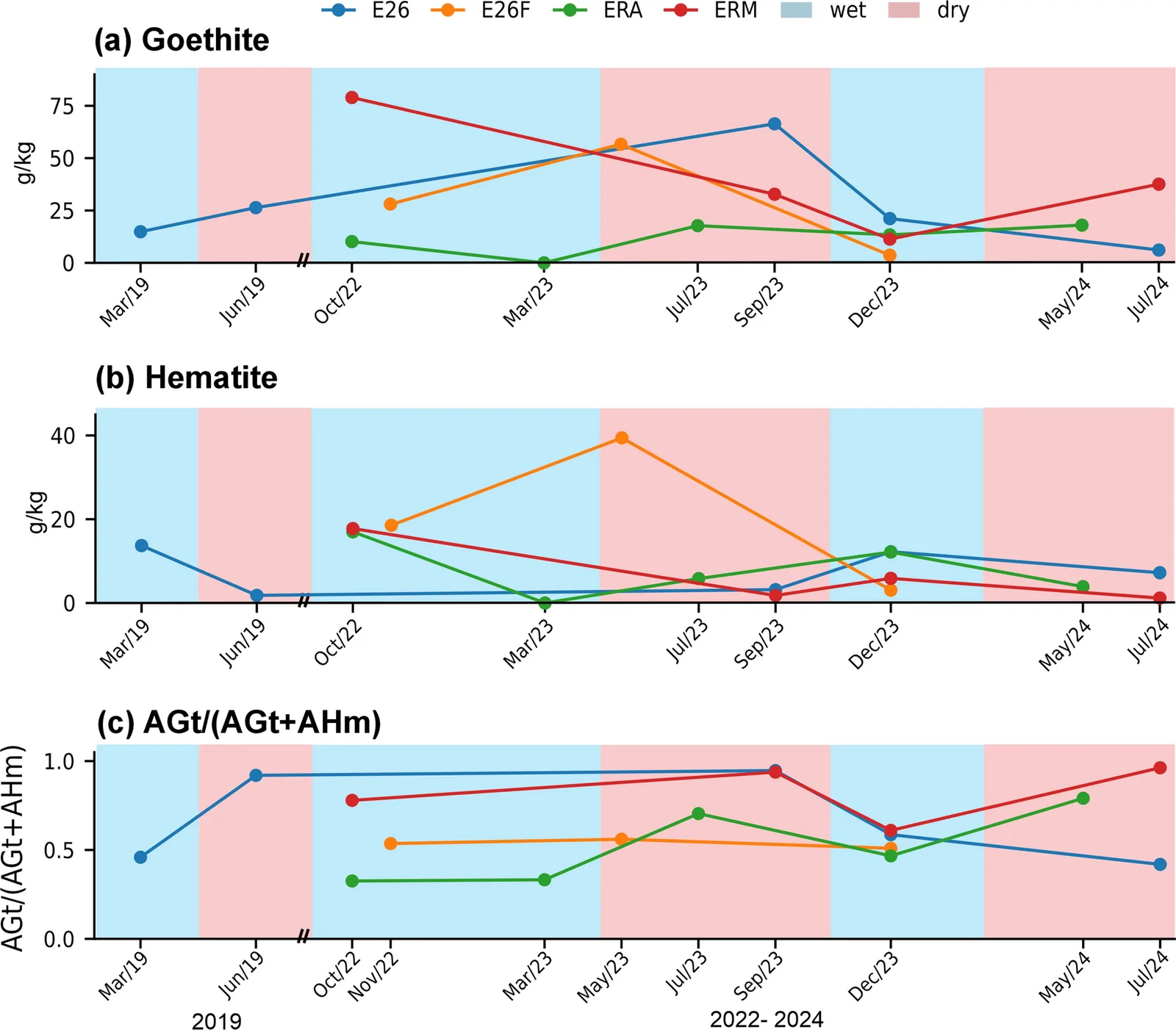
Seasonal and spatial dynamics of Fe oxyhydroxide assemblages in sediments of the lower Doce River basin. Variations in goethite (**a**), hematite (**b**), and the AGt/(AGt+AHm) ratio estimated from the relative intensities of goethite and hematite absorption features obtained by UV–Vis diffuse reflectance spectroscopy (**c**) illustrate shifts in the relative abundance of the dominant Fe oxyhydroxide phases under contrasting hydrological conditions. Blue and red shaded areas indicate wet and dry periods, respectively

The relative abundance of goethite and hematite also varied substantially among environments and monitoring periods (Fig. 4). Goethite concentrations ranged from near-zero values to approximately 80 g kg^−1^, with the highest contents recorded at ERM during Oct/22. In general, goethite predominated in most samples. Temporal fluctuations were more pronounced at the estuarine stations (E26 and E26F) and ERM, where goethite contents decreased markedly after Sep/23.

Hematite concentrations were generally lower than those of goethite and ranged from near-zero values to approximately 40 g kg^−1^. Elevated hematite contents were observed at E26F, with peak values occurring during May/23, whereas ERA and ERM consistently exhibited comparatively low concentrations throughout the monitoring period. Temporal variations in hematite were particularly evident at the estuarine stations, where increases during 2023 were followed by declines during subsequent campaigns.

The AGt/(AGt+AHm) ratio ranged from 0.33 to 0.96, indicating substantial variability in the relative contribution of both Fe oxyhydroxides. Higher values, reflecting goethite-dominated assemblages, were observed at E26 and ERM. Lower values occurred mainly during campaigns characterized by increased hematite abundance, particularly at E26F. Goethite remained the dominant Fe oxyhydroxide throughout most of the monitoring period, although the relative proportion of hematite increased at specific sites and sampling campaigns.

Mass-specific magnetic susceptibility values were consistently low across the mud sediments (Table 4). The χ_lf_ values ranged from 0.51 to 1.84 × 10^−6^ m^3^ kg^−1^, with the highest value recorded at E26 during Sep/23 and the lowest at ERA during Mar/23. Frequency-dependent susceptibility (χ_fd_) values ranged from 3.10 to 10.70%, with most samples remaining below 10%. The highest χ_fd_ value occurred at ERM during Sep/23, suggesting localized enrichment in finer magnetic fractions. The low χ_lf_ values indicate a predominance of antiferromagnetic Fe oxyhydroxides over ferrimagnetic minerals in the studied sediments.

### Integrated multivariate analysis of Fe oxyhydroxide mineralogy and PTE distribution

**Fig. 5:**
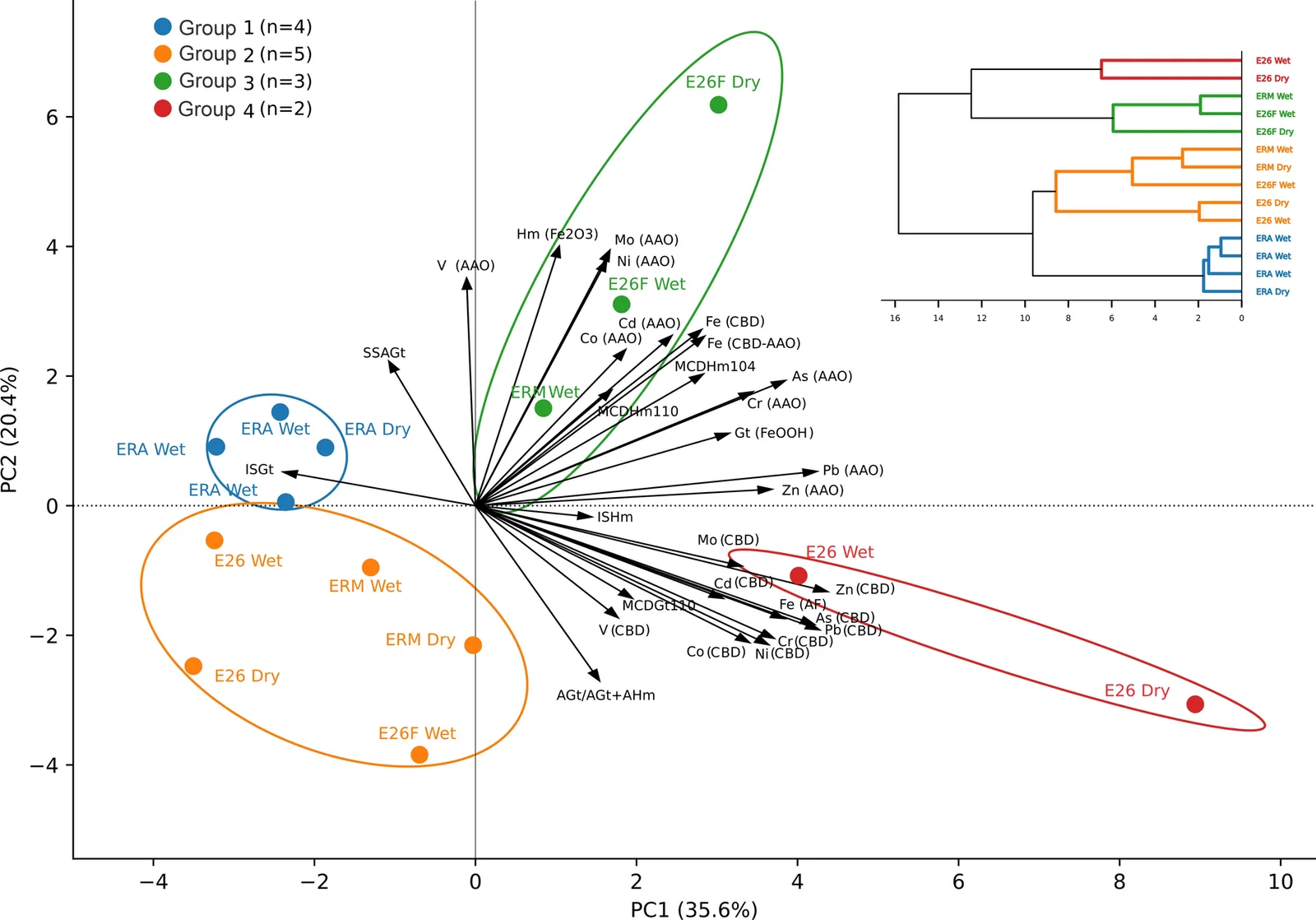
Integrated multivariate analysis of Fe oxyhydroxide fractions, crystallographic properties, and the investigated potentially toxic elements in sediments of the lower Doce River basin. PCA ordination and hierarchical clustering highlight spatial differences in mineralogical and geochemical associations among reservoir and estuarine environments

The first two principal components explained 56.0% of the total variance, with PC1 and PC2 accounting for 35.6% and 20.4%, respectively (Fig. 5). The ordination revealed a clear spatial separation among monitoring environments and was supported by hierarchical clustering, which identified four major sample groups.

Samples from ERA were clustered in the negative sector of PC1 and were associated primarily with goethite crystallographic attributes, particularly specific surface area (SSA_Gt_) and Al substitution (IS_Gt_). In contrast, estuarine samples from E26 and E26F occupied predominantly positive PC1 values and were closely associated with Fe oxyhydroxide fractions and PTE concentrations extracted by both CBD and AAO procedures.

Most PTEs, including As, Cr, Ni, Co, Pb, Zn, Cd, and Mo, exhibited strong positive loadings along PC1 and were closely aligned with Fe(CBD), Fe(AF), and hematite crystallographic parameters (MCDHm104 and MCDHm110). Variables associated with the AAO-extractable fraction, including Fe(AAO), Ni(AAO), V(AAO), and Mo(AAO), were preferentially distributed along positive PC2 values.

Samples from ERM occupied an intermediate position within the ordination space, whereas ERA, E26, and E26F formed more distinct clusters characterized by contrasting mineralogical and geochemical signatures. The hierarchical clustering analysis corroborated the PCA results by grouping samples with similar mineralogical and geochemical characteristics and separating reservoir and estuarine environments into distinct clusters.

These patterns indicate a strong association between Fe oxyhydroxides, particularly the CBD-extractable fraction, and PTE distribution across the monitored environments, whereas goethite crystallographic attributes were preferentially associated with reservoir sediments.

## Discussion

### Dynamic Fe cycling and mineralogical reorganization

The geochemical and mineralogical patterns observed along the lower Doce River are consistent with ongoing post-depositional transformation of the sedimentary Fe pool under fluctuating hydrodynamic and redox conditions. The temporal decoupling between Fe_d_- and Fe_o_-extractable Fe, together with the pronounced variability in Fe_o_/Fe_d_ ratios, indicates that Fe is not simply redistributed throughout the system but undergoes dynamic partitioning between operationally defined Fe fractions with contrasting degrees of crystallinity. Temporal fluctuations in Fe_d_ and Fe_o_ at estuarine stations, particularly E26F, are consistent with processes involving destabilization of crystalline Fe oxyhydroxides and formation of less crystalline or short-range-order Fe phases under alternating redox conditions. Similar processes have been reported in mining-impacted environments characterized by recurrent redox oscillations and strong hydrosedimentary variability (Kossoff et al., 2014; Queiroz et al., 2022).

The estuarine sector of the lower Doce River constitutes a highly reactive biogeochemical interface where salinity fluctuations, sediment resuspension, organic matter degradation, and seasonal hydrological forcing influence Fe oxyhydroxide stability and contaminant partitioning (Burdige & Komada, 2020; Queiroz et al., 2018; Sanders et al., 2015). Saltwater intrusion increases ionic strength and modifies mineral-solution interactions, affecting colloidal stability, aggregation behavior, and surface reactivity (Burdige & Komada, 2020; Queiroz et al., 2022). Simultaneously, organic matter degradation and restricted bottom-water circulation promote suboxic conditions capable of inducing partial reductive dissolution of Fe oxyhydroxides and the release of associated PTEs (Massario et al., 2020; Mermillod-Blondin et al., 2024; Queiroz et al., 2018; Schwertmann, 1991), while interactions between organic matter and Fe oxyhydroxide surfaces may further influence mineral transformation pathways and contaminant retention (Yi et al., 2022). Subsequent oxidation events may favor the formation of reactive short-range-order Fe phases with enhanced sorptive capacity (Burdige & Komada, 2020; Zhang et al., 2019). These coupled processes provide a plausible process-based framework for interpreting the pronounced temporal variability observed in Fe_o_, Fe_o_/Fe_d_ ratios, and PTE partitioning at E26 and E26F.

The relatively homogeneous mineralogical composition observed throughout the monitored system indicates that the temporal variability detected in the sediments was not associated with major changes in the bulk mineral assemblage. Quartz, K-feldspar, 2:1 clay minerals, kaolinite, gibbsite, muscovite, and rutile were consistently identified across all environments and monitoring periods. In contrast, Fe oxyhydroxides exhibited substantially greater variability, particularly in the estuarine sector. The predominance of goethite throughout the system, combined with temporal fluctuations in hematite abundance and Fe partitioning, suggests that the most pronounced post-depositional mineralogical changes were associated with the Fe oxyhydroxide pool, especially at E26 and E26F.

Additional evidence consistent with ongoing mineralogical reorganization is provided by the crystallographic and spectroscopic data. Goethite remained the dominant Fe oxyhydroxide throughout most sampling periods, as indicated by both XRD and DRS. However, substantial temporal variability in goethite and hematite abundances (i.e., the AGt/(AGt+AHm) ratio) indicates that the relative contribution of these phases is not constant through time. The marked decline in AGt/(AGt+AHm) observed during specific campaigns, particularly at E26F, coincided with periods of increased hematite abundance and pronounced fluctuations in Fe partitioning. Although these changes do not necessarily imply direct transformation between goethite and hematite, they indicate continuous modification of Fe oxyhydroxide assemblages under changing environmental conditions.

The crystallographic properties of goethite further support this interpretation. The notable variability observed in MCD, SSA, and IS suggests that Fe oxyhydroxides are not preserved as static mineral phases inherited from the original tailings deposition. Variations in SSA_Gt_ and IS_Gt_, together with changes in crystal dimensions, indicate modifications in goethite crystallographic properties that may influence surface reactivity and are consistent with ongoing mineral reorganization (Arruda et al., 2025; Camêlo et al., 2024; Queiroz et al., 2022). Periods characterized by greater variability in these crystallographic attributes were generally associated with environments exhibiting stronger hydrological fluctuations and geochemical gradients, supporting an association between estuarine forcing and temporal variability in Fe oxyhydroxide properties (Queiroz et al., 2018, 2022).

The contrasting behavior of Fe and Mn is consistent with recurrent redox oscillations within the sedimentary system. Whereas Fe remained predominantly associated with oxyhydroxide fractions, Mn exhibited substantially greater variability, particularly in the estuarine sector and episodically at ERM, with pronounced changes in Mn_o_ at E26F. This pattern likely reflects the lower thermodynamic stability of Mn oxyhydroxides relative to Fe oxyhydroxides, which may favor earlier reductive dissolution and enhanced Mn mobility during seasonal redox fluctuations (Liu et al., 2022; Patrick & Jugsujinda, 1992; Shi et al., 2021).

The multivariate analysis provides independent support for these interpretations. The PCA revealed a strong association between Fe oxyhydroxide fractions and PTE distribution, while also distinguishing reservoir and estuarine environments according to their mineralogical and geochemical characteristics. Estuarine samples were preferentially associated with Fe_d_, Fe_t_, hematite crystallographic parameters, and several PTEs, whereas ERA was more strongly associated with goethite crystallographic attributes. ERM exhibited an intermediate mineralogical and geochemical signature within the ordination space. Thus, the combined selective dissolution, crystallographic, spectroscopic, and multivariate evidence indicates that Fe oxyhydroxides remain active geochemical components of the lower Doce River sediments, with their mineralogical and geochemical variability closely associated with contemporary estuarine conditions while retaining the mineralogical imprint inherited from the Fundão tailings. Comparable relationships between sediment–water interface processes, oxygen availability, sediment reactivity, and element cycling have also been reported in reservoir environments characterized by pronounced biogeochemical gradients (Mermillod-Blondin et al., 2024).

### Relationships between Fe oxyhydroxide properties and PTE partitioning

The observed relationships among Fe oxyhydroxide fractions, crystallographic properties, and concentrations of the investigated PTEs indicate a strong association between Fe mineralogy and trace-element distribution. The close association of Fe_d_ with several PTEs (As, Cr, Ni, Co, Pb, Zn, and Mo) and hematite crystallographic parameters contrasts with the stronger association of reservoir sediments with goethite crystallographic attributes, particularly SSA_Gt_ and IS_Gt_. These patterns indicate that spatial differences in PTE partitioning are closely linked to variations in Fe oxyhydroxide composition and crystallographic properties, rather than being explained by differences in total elemental concentrations alone.

The crystallographic variability observed in goethite and hematite further supports a relationship between Fe oxyhydroxide structural organization and the retention of the investigated PTEs. Variations in MCD, SSA, and IS are consistent with post-depositional modification under contemporary environmental conditions. Such structural changes may alter the density and accessibility of reactive sorption sites available for trace-element retention (Sun et al., 2023). The coupled variability of IS_Gt_ and SSA_Gt_ is consistent with previous studies showing that increasing Al substitution in goethite may inhibit crystal growth, reduce crystallite dimensions, and promote higher specific surface areas (Camêlo et al., 2024; Cornell & Schwertmann, 2003; Kämpf & Schwertmann, 1983; Schulze, 1984). These crystallographic characteristics may enhance mineral surface reactivity and increase the availability of sorption sites involved in the retention of oxyanions and transition metals through surface complexation processes (Cornell & Schwertmann, 2003; Kämpf & Schwertmann, 1983).

The relationships between Fe-bearing fractions and the investigated PTEs observed in both the selective dissolution data and PCA suggest an important role of the more crystalline Fe oxyhydroxide pool in PTE retention within the sedimentary system. Arsenic exhibited particularly strong associations with Fe and Cr, consistent with the well-established affinity of oxyanions for Fe oxyhydroxide surfaces through inner-sphere complexation, adsorption, and co-precipitation mechanisms (Arruda et al., 2025; Camêlo et al., 2024; Giménez et al., 2007; Jiang et al., 2013; Mamindy-Pajany et al., 2011). Similar mechanisms have also been described for Cr retention by Fe oxyhydroxides through adsorption and surface complexation processes (Wang et al., 2024). At the same time, changes in Fe_o_/Fe_d_ ratios indicate shifts in the relative contribution of operationally defined Fe fractions with contrasting degrees of crystallinity, which may influence PTE retention under changing environmental conditions (Frierdich & Catalano, 2012; Lopez-Adams et al., 2021).

Additional mineralogical evidence is provided by the morphology and relative abundance of Fe oxyhydroxides. The elevated MCD_Gt110_/MCD_Gt111_ ratios observed at several stations indicate preferential crystal growth along the [110] direction, resulting in elongated goethite morphologies. Although this feature cannot be interpreted as exclusive evidence of mining-derived material, its persistence throughout both reservoir and estuarine environments, together with the associated geochemical signatures, supports the preservation of Fe oxyhydroxide assemblages inherited from the Fundão tailings (Camêlo et al., 2024). At the same time, variations in AGt/(AGt+AHm), SSA_Gt_, and Fe_o_/Fe_d_ indicate that these assemblages are not static and remain subject to post-depositional modification under present-day environmental conditions.

Diffuse reflectance spectroscopy and magnetic susceptibility analyses further corroborate the predominance of goethite and hematite throughout the monitored system. The consistently low magnetic susceptibility values indicate the dominance of antiferromagnetic Fe oxyhydroxides over ferrimagnetic phases such as magnetite, while variations in goethite and hematite abundance reveal changes in their relative contribution to the Fe oxyhydroxide assemblage. Similar relationships between magnetic susceptibility and the spatial variability of goethite and hematite have been reported in highly weathered tropical environments, where magnetic properties provide useful proxies for Fe oxide distribution and pedogeochemical processes (Silva et al., 2020). Together, these results indicate that Fe oxyhydroxide crystallographic properties are closely associated with the partitioning of the investigated PTEs and may influence mineral surface reactivity across the lower Doce River fluvial–estuarine continuum.

### Estuarine forcing and long-term environmental implications

The lower Doce River estuary represents the terminal depositional environment for tailings-derived materials transported downstream since the Fundão dam collapse (Queiroz et al., 2018; Richard et al., 2020). More than a decade after the disaster, Fe oxyhydroxides remain major geochemical hosts of the investigated PTEs throughout the fluvial–estuarine system. However, the results indicate that these mineral phases cannot be regarded as environmentally inert repositories. Instead, their geochemical behavior continues to be influenced by contemporary estuarine processes, including seasonal hydrological variability, sediment resuspension, salinity fluctuations, and redox oscillations (Burdige & Komada, 2020; Queiroz et al., 2022; Sanders et al., 2015).

The persistence of the investigated PTEs associated with Fe-bearing fractions within the sedimentary system has important implications for understanding their potential redistribution under changing environmental conditions (Burdige & Komada, 2020). The long-term stability of these associations may vary according to the mineralogical characteristics of Fe phases and the binding mechanisms controlling trace-element retention (Wang et al., 2024). Fe oxyhydroxides are recognized as important sinks for trace elements through mechanisms such as adsorption, co-precipitation, and structural incorporation. However, periodic changes in redox conditions may alter the stability of these mineral phases and modify the partitioning of associated contaminants. As a consequence, the investigated PTEs associated with sedimentary phases may represent a dynamic geochemical reservoir capable of responding to environmental perturbations rather than a permanently immobilized pool.

These findings highlight the limitations of environmental assessments based exclusively on total elemental concentrations. Total inventories provide valuable information regarding elemental abundance, but they do not capture the partitioning of trace elements among mineral-associated fractions or their potential redistribution under changing environmental conditions (Burdige & Komada, 2020; Crawford et al., 2022; Queiroz et al., 2022). The relationships observed between Fe oxyhydroxide properties and the partitioning of the investigated PTEs indicate that mineralogical and geochemical variability is closely associated with trace-element distribution among sedimentary fractions (Cornell & Schwertmann, 2003; Shi et al., 2021). Accordingly, maintaining mineralogical characterization and selective extraction procedures within long-term monitoring frameworks is valuable for understanding trace-element partitioning beyond that provided by total elemental inventories alone.

The results also reinforce the importance of estuarine environments as active geochemical interfaces within river basins affected by mine tailings. Rather than functioning solely as depositional sinks, estuaries may promote the retention, redistribution, and potential remobilization of contaminants through a combination of hydrodynamic, biogeochemical, and mineralogical processes, particularly during periods of increased hydrological forcing and sediment disturbance (Burdige & Komada, 2020; Crawford et al., 2022; Sanders et al., 2015). This role becomes particularly relevant in systems subjected to recurrent climatic variability and changing hydrological regimes, where shifts in salinity, sediment transport, and redox conditions may substantially influence contaminant cycling (Liu et al., 2022; Queiroz et al., 2022).

From a broader perspective, the lower Doce River provides a valuable natural laboratory for understanding the long-term evolution of tailings-derived Fe oxyhydroxides in tropical fluvial–estuarine environments. The persistence of the mineralogical signature inherited from the Fundão tailings, coupled with evidence of ongoing geochemical modification, indicates that post-depositional transformation processes may continue for years to decades after initial deposition. This study adds to a growing body of evidence indicating that the environmental legacy of large-scale mining disasters extends beyond contaminant inventories and encompasses the continuous evolution of the mineral phases that influence contaminant retention and mobility (Kossoff et al., 2014; Leal Pacheco et al., 2025; Queiroz et al., 2022).

## Conclusion

This study indicates that Fe oxyhydroxides associated with the Fundão dam tailings remain major geochemical hosts of the investigated potentially toxic elements (PTEs) in sediments of the lower Doce River fluvial–estuarine system more than a decade after the disaster. The combined selective dissolution, mineralogical, spectroscopic, magnetic, and multivariate analyses revealed that these mineral phases are not environmentally inert and remain closely associated with the partitioning of the investigated PTEs within the fluvial–estuarine system.

Temporal variations in Fe partitioning, Fe_o_/Fe_d_ ratios, Fe oxyhydroxide assemblages, and crystallographic properties indicate ongoing mineralogical reorganization under contrasting environmental conditions along the lower Doce River fluvial–estuarine continuum. The persistence of goethite and hematite as the dominant Fe oxyhydroxides, coupled with marked variability in their relative abundance and crystallographic characteristics, indicates that the mineralogical imprint inherited from the tailings remains preserved while undergoing post-depositional modification under present-day environmental conditions.

The strong associations observed between Fe oxyhydroxides and several investigated PTEs, particularly As, Cr, Ni, Co, Pb, Zn, and Mo, indicate a close relationship between Fe mineralogy and trace-element partitioning among sedimentary Fe-bearing fractions. Variations in crystallographic attributes, including crystal size, specific surface area, and Al substitution, further suggest that changes in Fe oxyhydroxide properties may influence their interactions with the investigated PTEs and, consequently, their partitioning among sedimentary fractions.

Collectively, these findings show that the environmental legacy of the Fundão dam collapse extends beyond the persistence of PTE-bearing sediments and encompasses the long-term mineralogical evolution of Fe oxyhydroxides associated with trace-element partitioning. By integrating selective dissolution, Fe oxyhydroxide mineralogy, crystallographic characterization, and multivariate analysis, this study provides new insights into the long-term geochemical evolution of mining-impacted sediments in tropical fluvial–estuarine environments and highlights the value of incorporating mineralogical indicators into long-term environmental monitoring frameworks.

## Data Availability

No datasets were generated or analysed during the current study.

## Declarations

### AI

During the development of this manuscript, generative artificial intelligence tools (ChatGPT, OpenAI) were employed to assist with English translation and improve the clarity and style of the scientific writing. All AI-assisted content was subsequently reviewed and validated by the authors, who assume full responsibility for the accuracy and integrity of the final manuscript.
